# The role of oral anticoagulants in epistaxis

**DOI:** 10.1007/s00405-018-5043-z

**Published:** 2018-06-23

**Authors:** A. M. S. Buchberger, A. Baumann, F. Johnson, N. Peters, G. Piontek, K. Storck, A. Pickhard

**Affiliations:** 1 Department for Ear- Nose- and Throat, Head and Neck Surgery, University hospital Klinikum rechts der Isar, Technical University of Munich, Munich, Germany; 2 Department of Otolaryngology Head and Neck Surgery, Helios Amper-Klinikum Dachau, Krankenhausstraße 15, 85221 Dachau, Germany

**Keywords:** Epistaxis, Oral anticoagulants, New oral anticoagulants

## Abstract

**Purpose:**

The purpose of this retrospective study was to identify the impact of oral anticoagulants on epistaxis with the focus on new oral anticoagulants.

**Methods:**

The study was conducted at the Department  for Ear- Nose- and Throat (ENT), Head and Neck Surgery, Technical University Munich, Germany. All patients presenting in 2014 with the diagnosis of epistaxis to a specialized ENT accident and emergency department were identified and analyzed in clinical data and medication.

**Results:**

600 adult cases, with a median age of 66.6 years were identified with active bleeding. 66.8% of all cases were anticoagulated. Classic oral anticoagulants (COAC) were three times more common in patients than new-generation oral anticoagulants (NOAC). Recurrent bleeding was significantly associated with oral anticoagulants (OAC) (*p* = 0.014) and bleeding location was most often anterior (*p* = 0.006). In contrast, severe cases, which required surgery or embolization were significantly more likely in non-anticoagulated middle-aged patients with posterior bleedings (*p* < 0.05). In our epistaxis cohort, OAC were highly overrepresented (40%) when compared to the general German population (1%) but COAC as well as NOAC played only a minor role in severe courses of epistaxis.

**Conclusion:**

Oral anticoagulation, especially with new-generation drugs, is not associated with more complicated and severe courses of epistaxis, but rather with recurrent bleeding. One should keep this information in mind when triaging the patient in the emergency room and when planning further procedures.

## Introduction

Epistaxis is common and occurs with a lifetime prevalence of 60% [1]. Even though only 6–10% of nosebleeds require the treatment of a doctor, it is, along with pharyngitis, the most common cause for ear–nose–throat (ENT) emergency visits [2, 3]. Of these, about 1.6/10,000 patients need to be admitted to the ward [1]. Depending on the localization of the bleeding, ENT doctors differentiate anterior (90%) from posterior (6–10%) epistaxis [4]. Generally, posterior epistaxis is considered more serious. The incidence of bleeding with classical oral anticoagulation has been reported between 10–17% for all events and 2–5% for severe events [5, 6].

New oral anticoagulants (NOAC) such as Rivaroxaban have been widely discussed in the last few years. Since NOACs showed an increase in profits of 69% between 2013 and 2014 and are increasingly prescribed [7], there is an increasing number of studies concerning safety of this new group of medications. Studies in the field of gastroenterology suggest that NOACs might cause an increased risk of bleeding due to the lack of controllability and antidotes as well as renal clearance deficiencies in patients > 75 years old [8, 9].

These findings raise an important question: do new oral anticoagulants promote epistaxis or do patients with (new) oral anticoagulants tend to have more severe disease with dramatic blood loss, need for repacking, surgery or for interventional radiology? Is a preexisting medication with an (new) oral anticoagulant an indicator or predictor for a more serious course of disease in nosebleeds? And should those patients be treated differently?

The following retrospective study analyzed epistaxis cases which presented during 2014 to a large ENT walk-in clinic of a university hospital and aimed to address the question posed above, and to provide a risk assessment for NOACs and possibly revise the triage and treatment guidelines concerning nasal bleeds. Since especially young doctors in the first years of their residency are the primary therapy providers, identifying predictors and revising triage and treatment guidelines will not only assist to their education but add to patient safety in minimizing errors in everyday practice.

## Methods

In this study, all patients visiting the ENT department of a large university hospital in 2014 with epistaxis were included. Patients seeking emergency help in the outpatient walk-in clinic as well as patients admitted to the ward were analyzed. The ENT clinic runs a 24-h seven-days-a-week emergency service as well as a walk-in outpatient clinic dedicated to provide emergency treatment. Patients’ age, sex, possible reason for the bleeding (trauma, spontaneous), localization of the bleed, recurring bleeds, underlying disease, medication with anticoagulants, blood pressure at the time of presentation, therapeutic approach, complicated courses and blood work (coagulation tests, hemoglobin level, platelet count) were collected. Recurrent bleeding episodes of the same patient cohort up to April 2018 were additionally registered. This was done in an anonymized way according to the data privacy protection law and in agreement to our Ethics committee (90/16 S). Patients below the age of 18 were not included in this study. The working hypothesis of this study was that patients with a preexisting medication of (new) oral anticoagulants experience more often complications or have more serious disease courses when presenting with epistaxis at the ENT emergency service. If this hypothesis proves true, preexisting anticoagulant medication could be used as a predicting factor and could assist in the triage of patients at presentation as well as during treatment. Additionally, this study will attempt to address whether new oral anticoagulants lead to more severe or prolonged bleedings compared to classic oral anticoagulants. An additional goal was to determine a risk assessment for this new group of medication, which as of yet has not been published for epistaxis.

After all data were collected using written patient data sheets as well as SAP (Systems, Applications & Products in Data Processing)-based house internal documents, statistical analysis was performed using SPSS 23.0 software (IBM SPSS Software from Sievers-group), provided by the Technical University of Munich. First, a descriptive statistic was performed to characterize the patient cohort. To compare the status of medication with anti-coagulants (yes/no, which kind) and complicated courses, the Chi-square test was performed. Complicated courses were defined as cases with relapses after initial therapy, surgical intervention, or the need for interventional radiology. For admitted patients, complicated courses were additionally defined based on the length of stay. Patients with hospital stays shorter than 4 days were considered less serious cases than patients who needed to stay 4 days or longer. Additionally, complicated courses were then analyzed within, separately looking at relapses and severe courses (surgery, embolization and length of stay > 4 days). To assess the risk of recurrent bleeding under anticoagulation further, we followed up the cohort until April 2018 to search for further timely independent relapses and checked for correlations between recurrent bleeding and choice of treatment or location of the bleeding.

To adjust for potential confounding variables such as age, sex, underlying disease, or location of the bleeding, and to take into account data clustering, a random effects logistical regression model was fitted. For a proper statistical analysis, guidance was given by the Institute for Medical Statistics and Epidemiology of the Technical University Munich.

## Results

### Descriptive analysis of the patient cohort

769 patients presented in 2014 to the ENT emergency department with the diagnosis of epistaxis. 149 patients showed no signs of active bleeding nor was the site of bleeding detectable. Therefore, these patients were excluded in the following statistics.

**Fig. 1:**
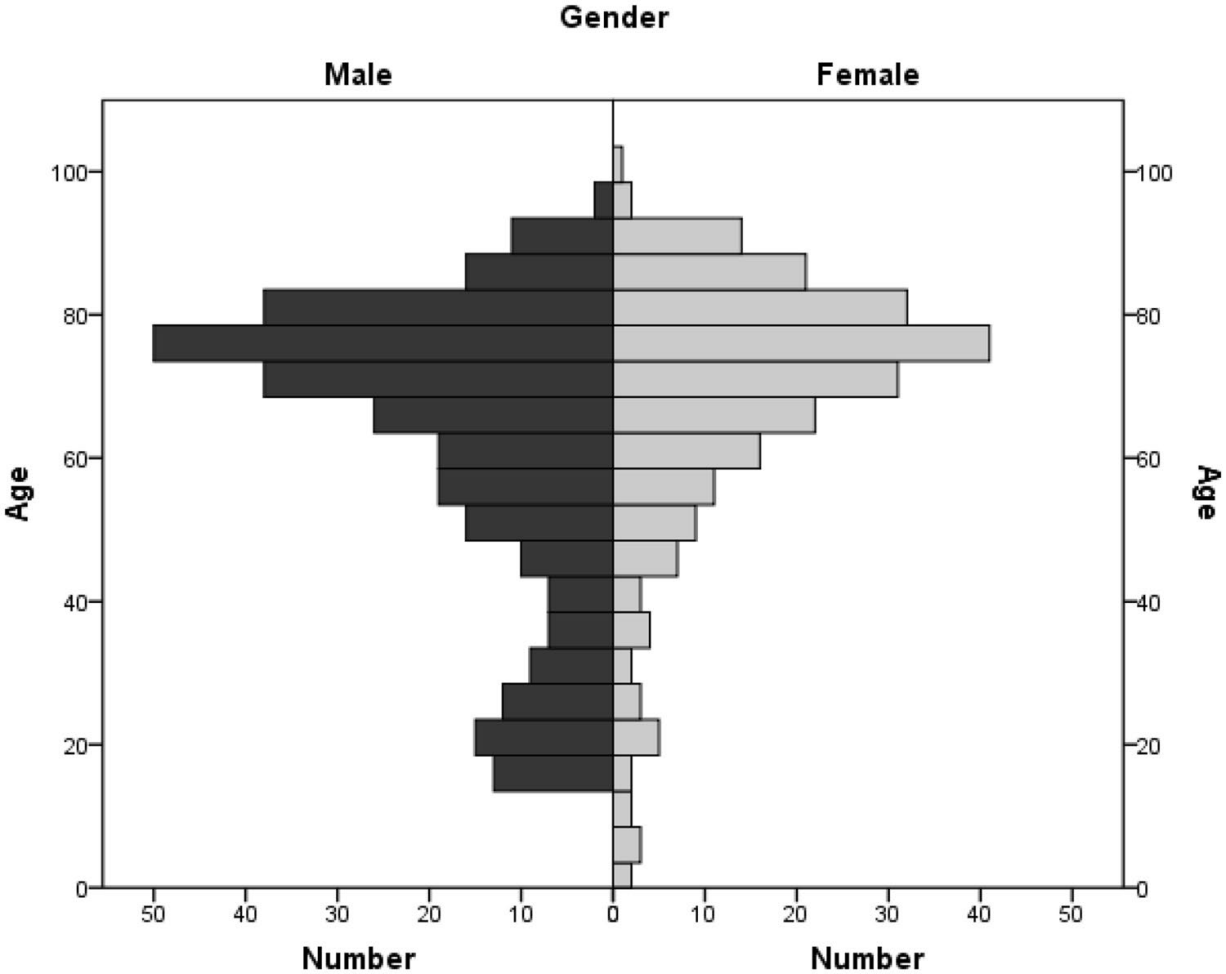
Age distribution of the epistaxis cohort (*n* = 541). Median age 64.2. Gender distribution: male 57.3%; female 42.7%. Median age < 18 years: 12.3

541 patients presented with a detectable bleed and were thus included in this study. The medium age at presentation was 64.2 ± 20.38 years and age distribution can be seen in Fig. 1. 327 patients were male (57.3%) while 243 patients were female (42.7%). 20 patients were below the age of 18 with a peak at the age of 15–17 (12 patients = 60%). This patient group was also excluded from the following statistics.

Thus, a cohort of 521 patients, respectively, 600 cases (due to 79 multiple presentations) above the age of 18, presenting with active nosebleeds was analyzed.

#### Causes and locations of epistaxis

Of these 600 cases, 7.8% presented with relapsing nasal bleeds. Secondary epistaxis was found in 13.5% of the bleeds due to a trauma and 20.8% of the cases presented with hypertensive blood pressures above 140/90 or in 64 cases even hypertensive urgency, defined as blood pressures above 180/120 mmHg as defined by the European Society of Hypertension and the European Society of Cardiology. In 57.8% of all cases, the treating doctor was not able to discover the reason for the bleeding.

Most bleedings were identified as anterior (72.7%), while only 5.8% were attributable to the posterior location. 6.5% of cases described a diffuse bleeding from the turbinates. 1.3% of all cases presented with multifocal bleedings due to telangiectasis in Osler’s disease. Only in 13.7% of all cases the treating physician was not able to clearly identify the source of bleeding (Table 1).

**Table 1:** Epistaxis cohort for 2014 above the age of 18 with descriptive statistics

	All cases > 18	Outpatient	Inpatient
Number of cases	600	510	90
Median age in years	66.6	66.0	69.7
Gender			
Female	256	215	41
Male	344	295	49
Female:Male	1:1.3	1:1.4	1:1.2
Cause of bleeding			
Trauma	(18) 13.5%	(72) 14.1%	(9) 10%
Hypertensive emergency	(125) 20.8%	(105) 20.6%	(20) 22.2%
Relapse	(47) 7.8%	(25) 4.9%	(22) 24.4%
Unknown	(347) 57.8%	(308) 60.4%	(39) 43.3%
Location of bleeding			
Anterior	(436) 72.7%	(402) 78.8%	(34) 37.8%
Posterior	(35) 5.8%	(13) 2.5%	(22) 24.4%
Conchae nasales	(39) 6.5%	(30) 5.9%	(9) 10.0%
Telangiectasis	(8) 1.3%	(5) 1%	(3) 3.3%
Unknown	(82) 13.7%	(60) 11.8%	(22) 24.4%
Site of bleeding			
Left	(290) 48.3%	(241) 47.3%	(49) 54.4%
Right	(263) 43.8%	(227) 44.5%	(36) 40%
Both sides	(36) 6%	(31) 6.1%	(5) 5.6%
Unknown	(11) 1.8%	(11) 2.2%	–
Therapy			
Conservative	(23) 3.8%	(21) 4.1%	(2) 2.2%
Bipolar coagulation	(409) 68.2%	(382) 74.9%	(27) 30.0%
Anterior packing	(138) 23%	(99) 19.4%	(39) 43.3%
Posterior packing	(7) 1.2%	(2) 0.4%	(5) 5.6%
Surgery (i.e. ligation of sphenopalatine artery)	(16) 2.7%	(1) 0.2%	(15) 16.7%
Interventional embolization	(2) 0.3%	–	(2) 2.2%
Severity of bleeding			
Hb relevant	(62) 10.3%	(24) 4.7%	(38) 42.2%
< 0.5 mg/dl	(538) 89.7%	(486) 95.3%	(52) 57.7%
0.5–0.9	(25) 4.2%	(14) 2.7%	(11) 12.2%
1.0–1.9	(24) 4%	(7) 1.4%	(17) 18.9%
2.0–2.9	(6) 1%	–	(6) 6.7%
3.0–3.9	(4) 0.7%	(2) 0.4%	(2) 2.2%
4.0–4.9	(1) 0.2%	–	(1) 1.1%
> 5.0	(2) 0.2%	(1) 0.2%	(1) 1.1%
Deranged coagulation			
–	(139) 23.2%	(102) 20%	(37) 41.1%
Quick/INR	(11) 1.8%	(5) 1%	(6) 6.75%
aPTT	(96) 16%	(79) 15.5%	(17) 18.9%
Thrombocytes	(4) 0.7%	–	(4) 4.4%
aPTT&INR	(27) 4.5%	(18) 3.5%	(9) 10%
Anticoagulation			
	(401) 66.8%	(344) 67.5%	(57) 63.3%
NOAC	(43) 7.2%	(34) 6.7%	(9) 10%
COAC (Phenprocoumon)	(138) 23%	(116) 22.7%	(22) 24.4%
1 PAI	(100) 16.7%	(89) 17.5%	(11) 12.2%
Heparinoids	(9) 1.5%	(8) 1.6%	(1) 1.1%
2 PAI	(38) 6.3%	(37) 7.3%	(1) 1.1%
NOAC + 1 PAI	(16) 2.7%	(11) 2.2%	(5) 5.6%
COAC + 1 PAI	(41) 6.8%	(37) 7.3%	(4) 4.4%
COAC + 2 PAI	(11) 1.8%	(8) 1.6%	(3) 3.3%
NOAC + COAC	(3) 0.5%	(2) 0.4%	(1) 1.1%
NOAC + COAC + 2 PAI	(2) 0.3%	(2) 0.4%	–
Complicated/serious courses			
	(77) 12.8%	(25) 4.9%	(52) 57.8%
Relapses	(47) 7.8%	(25) 4.9%	(22) 24.4%
Surgery	(16) 2.7%	(1) 0.2%	(15) 16.7%
Embolization	(2) 0.3%	–	(2) 2.2%
Admission ≥ 4 nights	(47) 7.8%	–	(47) 52.2%

#### Patient intake and blood examination

15% of all cases had to be admitted to the ward due to the severity of bleeding or multimorbidity of the patient with a medium stay of 3.8 nights. Recurrent bleeding was a prominent reason for admission and was responsible for 24.4% of all admitted cases. Identified posterior epistaxis patients were admitted in 62.9% of cases, while anterior epistaxis cases were admitted in 7.8% of the cases. The location of the bleeding was significantly associated with admission to the ward (*p* < 0.001), reflecting the severity of posterior epistaxis.

In more severe cases, or when patients were anticoagulated or reported about severe bleeding, blood work was performed. Of 371 cases, 25.7% showed a hemoglobin (Hb) level below the standard range while 6.5% had Hb levels below 10 mg/dl. Hb-relevant epistaxis with a drop > 0.5 mg/dl occurred in 10.3% of all cases while a drop above 2 mg/dl was detected in 2.7%. For patients admitted to the ward, 42.2% showed Hb levels below the standard range with 14.4% below 10 mg/dl. Hb drops in the course of stay were detected in 43.3% with 11.1% showing severe drops of more than 2 mg/dl.

Coagulation parameters were analyzed in 321 cases and ranges of coagulation times were adjusted accordingly if anticoagulant medication was taken. They revealed deranged parameters of either activated partial thromboplastin time (aPTT), international normalized ratio (INR) or in thrombocyte numbers in 23.3% of all cases and 41.1% of admitted patients. A deranged aPTT was most often seen (68.6%) in these cases but could not exclusively be explained by the medication (Table 1).

#### Treatment of epistaxis

Most commonly, epistaxis was stopped using bipolar coagulation (68.2%), followed by anterior packing. Posterior packing as exclusive treatment was only necessary in 1.2% of all cases, and in 5.6% of the admitted cases, respectively. 2.7% of all cases had to go to the operating room, most commonly this involved ligation or coagulation of the sphenopalatine artery in cases with posterior bleeding. In 0.3% it was necessary to involve the interventional radiology to embolize vessels. Conservative methods, such as nasal ointments or applying vasoconstrictory medication such as naphazoline together with the application of pressure to the nasal alae, were successful in 3.8% of all cases. Details and further information are displayed in Table 1.

### Anticoagulant medication and epistaxis

66.8% of all epistaxis cases were anticoagulated. The percentage of anticoagulant medication increased with age (Table 2).

**Table 2:** Age, percentage of admission, length of stay, percentage of severe courses, hb-relevant bleedings, relapses and successful therapy compared between the distinct groups of anticoagulants and non-anticoagulated patients

Medication	Median age in years	Admission (*n* = x) % OR [95% CI] *p* value	Median number of nights	Severe courses (*n* = x) % OR [95% CI] *p* value	Hb-relevant bleedings (*n* = x) % OR [95% CI] *p* value	Relapses (*n* = x) % OR [95% CI] *p* value
None (*n* = 199)	53.7	(33) 16.6%	4.1	(24) 12.1%	(16) 8%	(17) 8.5%
NOAC (*n* = 43)	77.9▲	(9) 20.9%▲ *1.332* [0.8–3.04] *0.496*	2.3▼	(2) 4.7%▼ *0.356* [0.81–1.57] *0.172*	(4) 9.3%▲ *1.000* [0.31–3.28] *1.000*	(6) 14.0%▲ *1.736* [0.64–4.70] *0.277*
COAC (*n* = 138)	74.4▲	(22) 15.9%▼ *0.954* [0.53–1.72] *0.876*	3.4▼	(10) 7.3%▼ *0.570* [0.17–1.05] *0.492*	(17) 12.3%▲ *1.137* [0.54–2.39] *0.736*	(13) 9.4%▲ *1.113* [0.52–2.37] *0.781*
PAI (*n* = 100)	71.2▲	(11) 11.0%▼ *0.622* [0.30–1.29] *0.202*	4.4▲	(5) 5.0%▼ *0.384* [0.14–1.04] *0.059*	(11) 11.0%▲ *1.437* [0.62–3.36] *0.401*	(11) 11.0%▲ *1.323* [0.60–2.94] *0.492*
Combination (*n* = 111)	71.1▲ (± 13.362)	(14) 12.6%▼ *0.726* [0.37–1.42] *0.351*	4.4▲	(6) 5.4%▼ *0.417* [0.17–1.05] *0.064*	(13) 11.7%▲ *1.246* [0.56–2.78] *0.591*	(31) 27.9%▲ *4.149* [2.17–7.93] < *0.0001*

Odds ratios and *p* values are in relation to the group without medication. The inverted triangles ▲▼ indicate increases/decreases in comparison to patients without medication

*CI* confidence interval

24.5% of all cases and 36.7% of the anticoagulated patients took a classic oral anticoagulant. For our cohort, in 96% this was Phenprocoumon, followed by Heparin (1.5%) and Enoxaparin (1.5%) and others (Tinzaparin, Mono Embolex and Hirudin each 0.5%). Only 7.2% took an oral anticoagulant of the new generation on the other hand, mostly Rivaroxaban (92%), followed by Apixaban (4.7%) and Dabigatran (3.1%). The ratio of COACS to NOACS was, therefore, 3.2:1. 16.7% of our cohort were anticoagulated with platelet aggregation inhibitors, in 97% ASS, followed by Clopidogrel (3.4%). 18.5% took more than one anticoagulant in different combinations (Table 1). For admitted patients, the preexisting medication with an anticoagulant was less when compared to the overall cohort (63.3%) but the ratio of NOACs was with 10% higher than compared to the ambulatory cohort (6.7%).

Medication with anticoagulants alone was no reason for the admission to the ward (*p* = 0.467) in general and also for the distinct groups of medication (*p* > 0.2). Adapted to age, admission to the floor differed only significantly for patients of the platelet aggregation inhibitors (PAI) group, who were less often admitted (OR 0.460, 95% CI 0.213–0.994, *p* = 0.048). The unbalanced coagulation parameters in the blood work were, as suspected, connected to the anticoagulation medication (*p* < 0.001).

### Serious/complicated courses

We defined cases as complicated or serious courses, if they presented with recurrent epistaxis shortly after initial treatment with the necessity of re-assessment and retreatment or severe cases that had to undergo surgery or interventional radiology. Also included were cases with prolonged stay above the average of 4 nights. 12.8% of all cases were affected by these serious courses with 61% having recurrent epistaxis, 21% surgery, 3% embolizations and 61% prolonged stays.

Severe courses were most commonly found in the group between the ages of 40–59 years (*p* = 0.003) with equal gender distribution and in patients with posterior bleeding (*p* < 0.001) or both (*p* = 0.012). They were additionally associated with frequent relapses (*p* < 0.001). Non-anterior bleeding was also associated with Hb-relevant bleeding (*p* < 0.001), and posterior bleeding showed the most pronounced drops of Hb levels of all groups while these patients were significantly less often anticoagulated (*p* = 0.006) than older epistaxis patients of other locations.

Anticoagulated patients showed shorter durations of stays when admitted to the floor (*p* < 0.02). They also showed less serious courses (*p* = 0.009, OR 0.46, 95% CI 0.256–0.84) and more recurrent bleedings without the need for surgical or radiological interventions (*p* = 0.014, OR 1.9, 95% CI 1.09–3.39). The group taking combinations of anticoagulant medication most frequently showed recurrent bleeding episodes (27.9%), while COAC relapsed in 9.4% and NOAC in 14%. For the combined medication group, especially combinations with PAI seemed to promote relapses (Table 2).

#### Long-term bleeding relapses in relation to the anticoagulation status

To assess whether patients with anticoagulant medication had recurrent bleeding not only in the short term but also with a prolonged follow-up time, the patient cohort (521 patients) was followed up until April 2018. We identified 52 cases of recurrent bleeding with 40 cases under oral anticoagulation. We found a trend towards a higher risk of recurrent bleeding episodes for anticoagulated patients as compared to patients with no anticoagulant medication (odds ratio of 2.035, CI 0.91–4.548) which did not gain statistical significance (*p* = 0.79). Patients with COACs seemed to have a higher risk compared to patients with NOACs although this trend did also not reach significance. Only patients with a combination of anticoagulant medication showed a statistically significant higher risk for recurrent bleeding episodes (*p* = 0.012), which confirmed our short-time findings. These recurrent bleedings showed no correlation either with the choice of treatment of the first episode or with the initial location of bleeding documented in 2014.

### Confounding variables and epistaxis

**Fig. 2:**
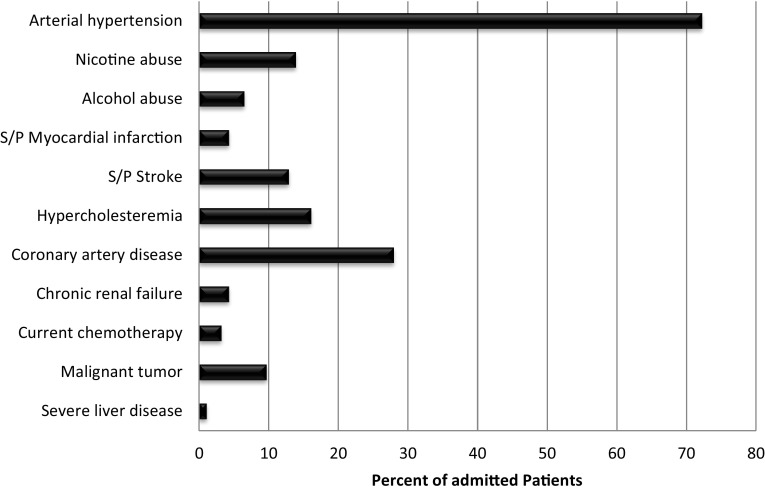
Underlying disease and risk factors for epistaxis of the in-patient cohort (*n* = 90). 77.8% of all admitted patients presented with ≥ 1 preexisting diseases

In the group of admitted patients, comorbidities were well documented, with 77.8% of these patients presenting with one or more underlying diseases potentially increasing the risk of bleeding. These comorbidities were not associated with severe courses (*p* = 0.225), however. 26.7% of admitted patients presented with one comorbidity while 51.1% had multiple comorbidities. Preexisting arterial hypertension (defined using the WHO criteria of blood pressure > 140/90 mmHg and patients treated with antihypertensive medication) was found in 72.2% of all admitted cases. Although patients with hypertension tended to be admitted to the floor more often, this trend was not significant (*p* = 0.06) and was again not associated with severe courses (*p* = 0.067). The second most common comorbidity was coronary heart disease. This reflects the high number of anticoagulated patients in this cohort. All comorbidities documented in patient charts as seen in Fig. 2 have been associated with an increased risk of nasal bleeds.

## Discussion

### Simple anterior nasal bleeding was the most common as expected

A comparison of gender distribution with other studies illustrates that our cohort is gender representative of patients presenting with epistaxis [10]. However, in the patient group under 18 years a surprising peak at the age of 15–17 (12 patients = 60%) was seen which is not in concordance with the internationally published two peak age distributions with a first peak around the age of 10 [3].

Reasons for epistaxis, including trauma, hypertensive emergency, or relapse [11, 12] as well as the common localization of the bleeding in the anterior region in our cohort were also comparable with other studies [4, 13, 14]. The number of patients admitted to the ward was similar to other studies, as was the proportion of comorbidities or reasons for severe bleeding [15]. Similar to Soyka et al., epistaxis was most commonly stopped with bipolar coagulation, followed by anterior packing [16].

### Epistaxis patients were above average often anticoagulated

In our cohort, 66.8% of all epistaxis cases were anticoagulated which is a very high number compared to an estimated 1% in the general population [17] and up to 17% in other studies published elsewhere [5, 6]. But increased coagulopathies in epistaxis patients have been described before [10]. The increased rate of anticoagulant medication correlated with age similar to Bernmüller et al. [18].

In concordance with Smith et al. [19], 24.5% of all cases and 36.7% of the anticoagulated patients, respectively, took a classic oral anticoagulant. The ratio of COACS to NOACS was, therefore, 3.2:1, which is higher than the nationwide ratio of 1.9:1 in DDD (Defined Daily Dosage) for Germany in 2014 [7]. The anticoagulation with platelet aggregation inhibitors was less than what Soyka et al. found in their cohort [20]. Unbalanced coagulation parameters were connected to the anticoagulation medication. With COACs, it was not surprising that in 5.8% of cases pathological INR values were detected. This is, however, significantly less than that published by other authors [19].

Preexisting arterial hypertension was seen as a confounder and has also been associated with epistaxis in multiple publications [10, 21, 22]. Our number is significantly higher compared to epidemiologic studies which demonstrated a 35–40% prevalence for people above the age of 35 although the steep increase of numbers with age has to be taken into account for our cohort [23].

### Patients with NOACs have no increased risk in nose bleeds

In summary, according to our data, elderly patients above the age of 60 presenting with epistaxis were less likely to exhibit posterior bleeding (OR 0.25, 95% CI 0.12–0.40, *p* = 0.0001) or to undergo severe courses (OR 0.44, 95% CI 0.26–0.75, *p* = 0.0026). Although they were most likely on anticoagulant medication (OR 11, 95% CI 8.83–15.22, *p* < 0.0001), they were more likely to have anterior bleeding, which was less likely to result in severe blood loss or prolonged hospital stays. This is partly in concordance with Bermüller et al. [18]. Anticoagulated patients were more likely to exhibit relapses than patients without anticoagulation, but these relapses did not tend to result in severe courses. In the group of anticoagulated patients those put on new oral anticoagulants such as Rivaroxaban, which was found most often as a NOAC in our cohort, did not show more severe courses or Hb-relevant bleedings or relapses than other anticoagulants although there was a not statistically significant trend towards recurrent bleeding as well as less serious courses. In our cohort, NOAC patients were admitted to the ward more often compared to patients on other anticoagulants. This might be due to their higher average age or due to caution towards this new group of medication. However, these patients were dismissed much earlier compared to the other groups.

Overall, since in our cohort NOACs were on the one hand underrepresented compared to nationwide prescription numbers but also showed few signs of severe bleeding, we have come to a positive judgment despite the current literature suggesting increased risks in elderly patients with compromised renal clearing rates [8, 9]. We conclude that patients who receive NOACs do not have an increased risk of epistaxis compared to other OACs and especially when compared to COACs. If they do present with epistaxis, no other treatment regime or special care has to be taken compared to patients with classic oral anticoagulants and the recommendations of Spielmann et al. should be followed [2, 24]. Depending on age, multimorbidities and suspected volume of blood loss, a standardized workup including blood work if necessary should be performed; determining the coagulation parameters is not a standardized necessity. In cases of severe bleeding with pronounced hemoglobin drops and high likelihood of either surgery or embolization, it is advisable to specifically test plasma levels of anti-factor Xa for patients with Rivaroxaban, Apixaban or Edoxaban, and anti-factor IIa levels in patients taking Dabigatran [25]. As of yet, only Dabigatran has an antidote (Idarucizumab), though other anticoagulant antidotes are likely to be developed in the future providing further treatment options. The decision to end anticoagulant medication or antagonize its effect due to surgery or embolization is an interdisciplinary decision between the ENT surgeon, interventional radiologist, the treating ENT specialist and the internist. Considering the patient’s comorbidities and individual risk factors and following established guidelines will highly improve the patient’s outcome [2, 25, 26].
